# Prognostic Outcomes of Cutaneous Squamous Cell Carcinoma in Solid Organ Transplant Recipients: A Retrospective Comparative Cohort Study

**DOI:** 10.3390/jcm12247619

**Published:** 2023-12-11

**Authors:** Rafael Salido-Vallejo, Lourdes Escribano-Castillo, Javier Antoñanzas, Claudia Roldán-Córdoba, Antonio Velez, Leyre Aguado-Gil

**Affiliations:** 1 Department of Dermatology, University Clinic of Navarra, 31008 Pamplona, Spain; jantonanzas@unav.es (J.A.); laguado@unav.es (L.A.-G.); 2 Department of Dermatology, Reina Sofía University Hospital, 14004 Córdoba, Spain; antonioj.velez.sspa@juntadeandalucia.es; 3 School of Medicine, University of Córdoba, 14004 Córdoba, Spain; lourdesec94@gmail.com (L.E.-C.); claudia.roldan.sspa@juntadeandalucia.es (C.R.-C.)

**Keywords:** immunosuppression, immunocompromised, solid organ transplant, cutaneous squamous cell carcinoma, prognosis, non-melanoma skin cancer, recurrence, metastasis

## Abstract

Introduction: Cutaneous squamous cell carcinoma (cSCC) is the second most common cutaneous neoplasm, and its incidence is on the rise. While most cSCCs have an excellent prognosis, certain risk factors, especially immunosuppression, have been associated with higher rates of local recurrence (LR), metastasis, and poor prognosis. This study aims to assess the risk factors for LR and metastasis development in cSCC among solid organ transplant recipients (SOTRs) and compare these rates with those in immunocompetent patients. Materials and Methods: A retrospective observational study included cSCC cases from the University Hospital Reina Sofía in Córdoba, Spain, between 2002 and 2019. Demographic, clinical, and histopathological data were collected. Local recurrence and metastasis rates were analyzed, along with progression-free survival. Univariate analyses were performed to identify prognostic factors in SOTRs. Results: Among 849 cSCC cases, we found higher rates of local recurrence and metastasis in tumors developed by SOTRs compared to those in immunocompetent individuals. However, no significant differences in local recurrence, metastasis, or progression-free survival were observed between the two groups. Risk factors for adverse outcomes in SOTRs included tumor size > 2 cm, depth > 4 mm, and a higher Clark level. A total of 34.4% of SOTRs developed a second primary cSCC during the follow-up. Conclusions: In our study, cSCCs in SOTRs did not exhibit statistically significant differences in the rates of adverse outcomes compared to immunocompetent patients. The prognosis of cSCCs in SOTRs may be more related to other tumor-dependent risk factors than to the immunosuppression status itself. Future studies are needed to refine risk stratification and follow-up protocols to ensure the optimal management of high-risk cSCC cases, particularly among immunosuppressed patients.

## 1. Introduction

Cutaneous squamous cell carcinoma (cSCC) constitutes the second most prevalent cutaneous neoplasm following basal cell carcinoma (BCC) [1]. Its incidence has been progressively rising in recent decades, with an anticipated continuation of this trend in the forthcoming years [2]. The majority of cSCCs exhibit a relatively indolent clinical course, and surgical intervention remains the primary therapeutic modality, characterized by a high rate of curative outcomes [3].

While cutaneous squamous cell carcinomas (cSCCs) generally have an excellent prognosis, there are several risk factors that have been shown to be associated with a higher rate of local recurrence (LR), metastasis, or disease-specific death [4].

A particularly high-risk population for the development of keratinocytic carcinomas includes immunosuppressed patients. This special population has both an increased rate of keratinocyte carcinomas and a poorer prognosis [5]. Depending on the cause of the patient’s immunosuppression, the incidence of cSCC can vary considerably. For instance, patients with chronic lymphocytic leukemia are 13 times more likely to develop skin cancer, with a cSCC prevalence estimated around 3.2% [6]. Patients with HIV tend to develop non-melanoma skin cancer at an earlier stage and with a worse prognosis [7]. Solid organ transplant recipients (SOTRs) represent a particularly high-risk subgroup, with estimated rates of cSCC occurrence between 65 and 200 times greater than in immunocompetent patients [8].

The cumulative incidence of cutaneous cancer in SOTR patients, 10 years post-transplant, varies by population, ranging from 10–15% in Europe to as high as 45% in Australia [9]. Although basal cell carcinoma (BCC) is much more common than cSCC in immunocompetent patients, this ratio is reversed to 1:3 in immunocompromised patients [10].

Cutaneous carcinomas in SOTRs arise from a complex multifactorial process influenced by exposure to ultraviolet radiation, the immunosuppressive regimen, and human papillomavirus infections. All these contributing factors culminate in a diminished immune surveillance, consequently elevating the risk of cancer development [11].

SOTRs have a higher incidence of keratinocytic carcinomas, along with a disease-related mortality that exceeds that of the general population by more than 20 times [12]. While some risk factors for the development of local recurrences or metastases in SOTRs have been described, studies continue to show discrepancies and significant heterogeneity [13,14,15], making it crucial to better characterize the risk of cSCC in this high-risk patient subgroup. Therefore, our hypothesis is based on the possibility that cSCC developed in SOTRs may exhibit a different prognosis compared to those that arise in immunocompetent patients.

The main objective of our study is to assess the risk factors associated with the development of metastases or local recurrences of cSCC in SOTRs and to compare these rates of local recurrences and metastases with those in immunocompetent patients.

## 2. Materials and Methods

### 2.1. Study Population

A longitudinal retrospective observational epidemiological study was conducted, encompassing all consecutive cases of primary cutaneous squamous cell carcinoma (cSCC) in patients treated at the University Hospital Reina Sofía in Córdoba (HURS) between 1 January 2002 and 31 December 2019. Exclusion criteria comprised patients with in situ or microinvasive cSCC, anogenital SCC, keratoacanthomas, eruptive squamous atypia, and basosquamous carcinomas, as well as patients with progeroid syndromes or other genetic syndromes predisposing to keratinocyte neoplasias, such as xeroderma pigmentosum, albinism, epidermolysis bullosa, Muir-Torre syndrome, or Ferguson-Smith type. Recurrent or initially metastatic cases were also excluded from the study.

### 2.2. Data Collection

Demographic, clinical, and histopathological data were retrieved from the Dermatology and Pathology Departments’ databases at HURS. The histopathological processing protocol for the biopsies was performed according to standard clinical practice and remained unchanged throughout the study period. All slides were assessed by expert dermatopathologists using hematoxylin and eosin staining. In cases where the diagnosis was uncertain or a more precise assessment was required (such as perineural invasion or lymphovascular invasion), relevant immunohistochemical stains were utilized.

The primary study variable was defined as the development of local recurrences or metastasis of cSCC in solid organ transplant recipients. Local recurrence (LR) was defined as the detection of biopsy-confirmed cSCC at the location of the prior excision. Metastasis was defined as the development of biopsy-confirmed cSCC either within the lymph nodes connected to the affected region or within a distant organ. The definition of progression-free survival (PFS) was the time from cSCC treatment to disease progression (LR or metastasis) or death from any cause.

Demographic and clinicopathological variables included age, gender, community type (rural or urban), personal history of skin cancer, cSCC location (head and neck, upper extremities, lower extremities, or trunk), time from cSCC diagnosis (months), clinical tumor diameter (cm), thickness (mm), Clark level (defined as infiltration of the tumor to: I epidermis; II papillary dermis; III papillary–reticular junction; IV reticular dermis; and V subcutaneous tissue), histologic differentiation (well-differentiated, moderately differentiated, poorly differentiated), tumor infiltration of deep structures (fascia, muscle, perichondrium/periosteum, cartilage or bone), presence of lymphovascular or perineural invasion, surgical margins status, development of local recurrence or metastasis, and type of metastasis (lymph node or distant metastasis). Perineural involvement was considered as clinically significant when the presence of tumor cells within the nerve sheath of a nerve located deeper than the dermis or measuring ≥0.1 mm was observed [13].

The standard surgical procedure performed in all cases was wide local excision, following the surgical margin guidelines specified in the European guidelines for cSCC treatment [14]. These guidelines specify peripheral clinical margins from 6 to 10 mm for high-risk to very-high-risk cSCC and 5 mm for low-risk cSCC. All patients included in the study underwent clinical monitoring, and in cases of high-risk cSCC, imaging tests were conducted following the recommendations of the Non-Melanoma Skin Cancer Multidisciplinary Tumor Board at HURS (for all cases included after the tumor board’s establishment in 2010) or by the patients’ physicians. Positive surgical margins were considered when the pathological report indicated the presence of residual tumor, its immediate proximity to the surgical edge, or when the distance to the margin was reported as less than 0.1 mm. We also collected data about the department that conducted the primary surgical treatment.

Data related to immunosuppression included patient age at the time of transplantation (years), the respective immunosuppressive drug regimen used, the presence of skin tumors after transplantation and the time to the development of the second tumor.

### 2.3. Statistical Analysis

Descriptive analysis of variables was performed, calculating the absolute and relative frequencies for qualitative variables and the mean, standard deviation, minimum, and maximum values for quantitative variables. A 95% confidence interval was estimated.

Parametric tests were employed for baseline group comparisons. The Kruskal–Wallis H test was used to compare ordinal qualitative variables across more than two groups, and chi-squared tests were performed for nominal qualitative variables. For quantitative variables, Student’s *t*-test for independent groups and analysis of variance were used for comparisons involving more than two groups.

Kaplan–Meier survival curves were generated for transplant patients with respect to the development of metastasis and local recurrence. Log-rank tests were then employed to assess survival differences between both groups.

A secondary analysis was conducted to evaluate risk factors for local recurrence and metastasis development in the immunocompromised cSCC subgroup following solid organ transplantation, along with the time from primary cSCC diagnosis to the occurrence of local recurrence or metastasis. The at-risk period for local recurrence or metastasis development was calculated from the date of primary cSCC diagnosis to the last review date, date of death, or the occurrence of local recurrence or metastasis.

Univariate analyses were performed to determine prognostic factors in the subgroup of cSCC cases in solid organ transplant recipients.

All tests were two-tailed, and significance was defined as *p* < 0.05. Data were collected, processed, and analyzed using SPSS v.17 statistical software (SPSS Inc, Chicago, IL, USA).

### 2.4. Ethical Considerations

The study complied with Good Clinical Practice guidelines, the principles of the Declaration of Helsinki, and Order SAS 439/2010 of 14 December, regulating the ethics committees for healthcare and biomedical research in Andalusia, Spain. Throughout the study, data confidentiality was maintained through coding to protect the identities of participants. The study was approved by the Ethics Committee of the University Hospital Reina Sofía in Córdoba (Protocol Code: RSV001.2.0).

## 3. Results

A total of 849 cutaneous squamous cell carcinomas from 64 SOTRs and 785 immunocompetent patients were included in the study. Demographic and clinicopathological characteristics of the study population are summarized in Table 1. The predominant sex was male among both SOTRs (81.2%) and immunocompetent patients (71.0%). The mean age at diagnosis was significantly lower in SOTRs (64.50 years, SD 12.62) compared to immunocompetent patients (79.71 years, SD 10.08) (*p* < 0.001).

Regarding the type of community, there was no significant difference between the groups, with a similar distribution of urban and rural patients. In terms of personal history of skin cancer, a higher percentage of SOTRs had a history of non-melanoma skin cancer (NMSC) compared to immunocompetent patients (65.6% vs. 48.5%, *p* = 0.02).

Among the group of SOTRs, the organ transplanted in most cases was the kidney (81.3%), followed by the heart (10.9%). As for tumor location, the head and neck region was the most common site for SCC in both groups, with a prevalence of 75% in SOTRs and 84.1% in immunocompetent patients.

Table 1 also presents several other clinicopathological characteristics of the primary cSCC, including the Clark level, histologic differentiation, perineural invasion, lymphovascular invasion, and the involvement of deep structures. No significant differences were observed between the tumors developed in SOTR and immunocompetent patients for these variables.

According to the variables related to the treatment of the cSCC, no statistical differences were observed in the surgical margin status, with 75% and 77.9% of SOTRs of immunocompetent patients, respectively, having negative margins. A small proportion of patients in both groups received adjuvant radiotherapy (SOTRs: 6.25%, immunocompetent patients: 5.2%, *p* > 0.05). The mean follow-up duration for our sample was 41.9 months (SD: 33.9), with no statistically significant differences between both groups (*p* > 0.05).

In our study, a total of 111 LR were identified, among which 10 occurred in SOTRs (10/64; 15.6%) and 101 in the immunocompetent group (101/785; 12.9%). The observed rates of metastasis in our study were 6.3% and 5% in the SOTR and immunocompetent groups, respectively. Among the four patients who developed metastasis in the immunosuppressed group, half presented with lymph node metastasis, while the other half had distant metastasis. In the immunocompetent group, the vast majority of metastases (38/39; 97.4%) were detected in the regional lymph nodes, with only one distant metastasis observed. When assessing the rates of LR and metastasis, no significant differences were observed between the two groups (*p* > 0.05).

**Figure 1:**
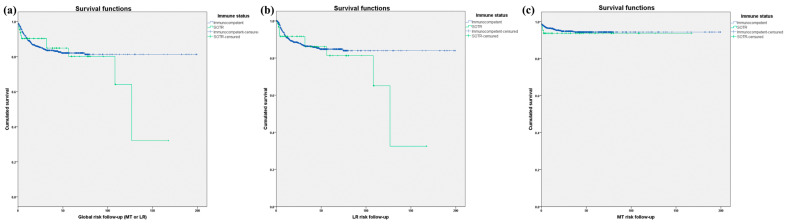
(a) Kaplan–Meier curves representing squamous cell carcinoma progression-free survival (metastasis or local recurrence) in solid organ transplant recipients and immunocompetent patients (Log-rank *x*^2^ = 0.277; *p* = 0.599). (**b**) Kaplan–Meier curves representing squamous cell carcinoma local recurrence-free survival in solid organ transplant recipients and immunocompetent patients. (Log-rank *x*^2^ = 0.716; *p* = 0.397). (**c**) Kaplan–Meier curves representing squamous cell carcinoma metastasis-free survival in solid organ transplant recipients and immunocompetent patients (Log-rank *x*^2^ = 0.307; *p* = 0.580).

We estimated the cumulative probability of developing metastasis or LR in cSCC in SOTRs in comparison to immunocompetent individuals (Table 2). At 6 months, the cumulative probability for LR was 8% for SOTRs and 5% for immunocompetent patients. This pattern continued at 12 and 24 months in the SOTRs group, having a slightly higher cumulative probabilities of LR in the immunocompetent patients. In terms of metastasis, 3% of SOTRs had metastasis at 6 months, 4% at 12 months, and 5% at 24 months, while immunocompetent patients displayed metastasis rates of 7% at each time point. No statistically significant differences were detected when comparing survival curves for the incidence of LR, metastasis, or PFS in patients stratified by their immune status (Figure 1).

Table 3 displays the results of univariate analyses using the Cox regression model for cSCC in transplant patients. Notably, a tumor diameter larger than 2 cm (HR 3.06, 95% CI 2.2–4.3, *p* = 0.001), a depth of invasion greater than 4 mm (HR 5.06, 95% CI 1.07–2.8, *p* = 0.040), and the Clark level of invasion (HR 3.7, 95% CI 1.3–10.4, *p* = 0.011) were associated with significantly higher relative risks of adverse outcomes in SOTRs. However, sex, age, SCC location, histologic differentiation, perineural invasion, lymphovascular invasion, and margin status did not show statistically significant associations with adverse outcomes.

A total of 22 patients from the 64 patients included in the SOTRs group developed a second primary skin tumor (22/64; 34.4%). The majority of these patients with multiple skin tumors were over 50 years old at the time of transplantation (20/22; 90.9%), and only three (13.6%) had undergone retransplantation. Among these patients, 95.5% initially presented with cSCC, while only 4.5% had BCC as their first cutaneous tumor. This trend persisted in the subsequent tumors that developed, with a higher frequency of cSCC (83.3%) compared to BCC (16.7%) as their second cutaneous tumor. The median number of cSCCs developed after transplantation was higher than the number of BCCs (median: 4 vs. 1; interquartile range: 4 vs. 5). The average time to the development of the second skin tumor among SOTR patients was 3.48 years (Table 4).

The most frequently used drugs in immunosuppressive regimens were tacrolimus (72.7%) and mycophenolate mofetil (40.9%), along with low-dose corticosteroids (90.9%). Inhibitors of the mammalian target of rapamycin (mTOR), including everolimus and sirolimus, were employed in 31.8% and 18.2% of patients, respectively. Overall, the various immunosuppressive regimens were consistently maintained for extended periods, with treatment durations averaging between 17.95 years for sirolimus and 4.41 years for mycophenolic acid (Table 5).

## 4. Discussion

In our population of 849 analyzed cSCC cases, we observed slightly higher rates of local recurrence and metastasis in SOTRs compared to immunocompetent patients, although no statistical differences were observed between the two groups. However, this remains a topic of debate because, while the immunosuppressed status of SOTRs has traditionally been considered a risk factor for worse prognosis in cSCC, the existing literature currently shows some controversy on this matter.

Several authors have suggested that immunosuppressed patients are at higher risk of experiencing poor outcomes (such as LR, metastasis, etc.) and specific disease-related mortality [15,16,17,18,19,20,21]. Tokez et al. [22], in a cancer registry study of over 10,000 patients, estimated the global risk of cSCC metastasis at 1.9%. This risk was significantly higher in immunosuppressed patients, especially in SOTRs, where up to 4% developed metastasis. Although most cSCC metastases occur within the first 2 years [16], SOTRs appear to have a latency period that can be longer, warranting extended follow-up in high-risk cSCC patients [23,24]. In a recent meta-analysis, the pooled risk of metastasis was significantly higher in SOTRs (7–11%) compared to immunocompetent patients (3.1–8.5%). However, the heterogeneity among the included studies requires cautious interpretation of the study’s conclusions [25]. Metastasis rates in our study did not significantly differ between SOTRs and immunocompetent patients, standing at 6.3% and 5%, respectively. These rates are higher than those observed in other population-based studies, where the overall rate of cSCC metastasis ranges from 1.8 to 1.9% [21,22]. However, the metastasis rates in our cSCC sample are slightly higher than those reported in cohort-based studies from reference hospitals, which vary from 3.1% to 5.15% [16,17,18,19,26] This circumstance is intriguing and might be explained by two possible reasons. The first might be that, as our center was a reference for other hospitals that refer high-risk cSCC cases to us but treat low-risk ones, it could have resulted in higher rates of poor outcomes. Additionally, the low absolute number of metastatic events makes the percentages less comparable with other observational study designs.

On the other hand, other authors have observed that the immunosuppressed state does not behave as an independent risk factor for the development of poor prognosis events in cSCC [24,27]. Recently, the group from Brigham and Women’s Hospital published a dual-center cohort of 8597 cSCC cases from 814 SOTRs and 4198 immunocompetent patients in which immunosuppression did not emerge as an independent risk factor for the development of metastasis or disease-specific death (DSD) after adjusting the analysis for tumor stage. However, they noted that patients had a greater number of tumors and presented higher T stages [28]. Therefore, the authors argued that SOTRs may have a worse prognosis not due to their immunosuppression per se but because of the development of tumors with a poorer prognosis that are treated at more advanced stages than in immunocompetent patients. In line with this, the work of Cheng et al. compared high-risk cSCC in immunocompetent and SOTRs, finding no significant prognostic differences between both groups [29]. Therefore, rather than the patient’s immunosuppression, what could influence the prognosis of SOTRs most is the stage of cSCC itself. Our study could support these findings, as we did not find prognostic differences between the cohort of cSCC in immunocompetent patients and SOTRs, which would be consistent with the notion that both groups do not differ in terms of poor prognosis outcomes. However, our low absolute number of poor outcomes in SOTRs could condition our results due to a limited statistical power. When evaluating this fact, it is important to consider that SOTRs are subject to more rigorous follow-up protocols than the general population, which could influence the early diagnosis of cSCC in our SOTR sample, explaining the absence of differences concerning other prognostic factors compared to tumors in immunocompetent patients.

Efforts have been made to identify specific risk factors in cSCC that may determine a higher risk of metastasis in SOTRs and whether these factors differ in comparison to immunocompetent patients. Genders et al. observed that factors such as the location on the head and neck, advanced age at transplantation, and the onset of the first cSCC were associated with a higher rate of metastasis [24]. Lanz et al. noted in a series of 51 SOTRs with aggressive cSCC that developed metastasis or disease-related death, these tumors were predominantly located in the head and neck, exhibited poor differentiation, had a depth greater than 6 mm and a clinical diameter exceeding 1.8 cm, and showed perineural invasion [30]. In our series, tumor size greater than 2 cm and parameters related to greater cSCC depth (depth > 4 mm and Clark level) were identified as risk factors for the development of poor outcomes in SOTRs, which aligns with the findings of the latter study. However, we have not found statistically significant differences concerning other highly relevant variables such as perineural infiltration or lymphovascular invasion, well-known risk factors in cSCC. This could be due to the infrequency of these histopathological risk factors, which necessitates a higher statistical power to more accurately assess the influence of these factors on the prognosis of SOTRs.

Apart from SOTRs being considered a high-risk population for developing a higher number of cSCCs with worse prognoses, some authors suggest that these tumors are more refractory to standard treatments. Consequently, cSCCs in SOTRs have shown a higher rate of local recurrence, metastasis, and disease-specific death following Mohs surgery, radiotherapy, and even the combination of both treatments [31,32,33].

As LR rates primarily depend on surgical margin status, it is much more common to experience LR in cases where there is a residual tumor [34,35]. In our study, we also did not observe statistically significant differences in terms of the LR rate between both groups. The percentage of affected margins in our sample was higher than in studies focused on Mohs surgery, likely because all patients were treated with wide local excision. However, since the rate of patients with R0 surgery did not vary between both groups, this could explain why there were no differences in the development of LR. Furthermore, not only could the presence of residual tumor in the surgical specimen be associated with a worse prognosis, but the distance to the surgical margin could also be relevant in this regard. Phillips et al. [36] analyzed the influence of this distance to the surgical edge in 92 patients undergoing advanced cSCC surgery, of whom 21 (22%) were immunocompromised. They observed that tumors removed with a microscopic margin greater than 5 mm showed improved disease-specific survival. However, the authors did not find a significant correlation between immunosuppression and margin distance concerning recurrence. In our study, we were unable to analyze this variable as it was not systematically documented in our center’s pathological reports, yet it undoubtedly represents a knowledge gap to explore in the future.

Our data revealed a higher prevalence of males in both cSCC developed in SOTRs and immunocompetent patients. This appears to be a general trend concerning cancer development and prognosis in men, wherein, globally, it tends to be more frequent and associated with poorer outcomes, although there is no clear evidence to explicitly elucidate this aspect. Specifically, for cSCC, the likelihood of occurrence in men is estimated to be up to three times higher than in women.

The type of transplanted organ also appears to have clinical relevance in estimating the risk of cSCC in SOTRs. Cardiotoracic transplant recipients have the highest incidence of cSCC, likely due to their more oncogenic immunosuppressive regimens, which often include azathioprine and voriconazole more frequently [37]. Renal transplant recipients have the second-highest incidence rate [38], which may be explained by their higher prevalence of actinic keratosis and the risk of progression to invasive cSCC. A recent study estimated that keratinocyte cancer mortality in kidney transplant recipients is 20 times higher than in the general population, with higher mortality observed in Caucasian males over 50 years of age [12]. In our study, over 90% of our patients received kidney and heart transplants, with immunosuppressive regimens primarily based on tacrolimus, mycophenolate mofetil, and low doses of corticosteroids. Azathioprine is the immunosuppressive drug most closely associated with the risk of developing cSCC, while calcineurin inhibitors (such as tacrolimus and cyclosporine), mycophenolate, belatacept, and mTOR inhibitors have not shown a clear increase in cSCC risk [39]. However, in renal transplant recipients, switching from calcineurin inhibitors to mTOR inhibitors has demonstrated clear benefits in reducing cSCC incidence and improving progression-free survival [9,40,41]. These new immunosuppressive regimens, along with other preventive measures adopted in SOTRs, could explain the declining trend in observed keratinocyte carcinoma incidences in these high-risk patients over the past 20 years and supports the role of the immunosuppression in the etiopathogenesis of skin cancer in SOTRs [42].

The annual risk of developing a second primary cSCC is significantly higher in SOTRs than in immunocompetent patients and increases in a monotonic fashion [38,39]. More than half of SOTRs develop a second primary cSCC [21], and 35% of them do so multiple times [5]. In our study, 35% of SOTRs developed a second primary keratinocyte cancer, with cSCC accounting for 95% of cases. The average time to development was 3.5 years, which is similar to the times observed in other studies. This higher incidence of cSCC has been suggested as one of the possible explanations to justify the worse prognosis observed in some studies among SOTRs. Developing more tumors at a faster rate might increase the likelihood of high-grade tumors, ultimately leading to a poor prognosis. According to this theory, the state of immunosuppression would have a more direct impact on carcinogenesis than on the immune response to the tumor. However, it is biologically more plausible that the patient’s immunological status affects both aspects.

These high incidence rates, along with the greater frequency of clinicopathological factors associated with poor prognosis in cSCCs developed by SOTRs, underscore the importance of implementing measures to reduce the occurrence of new skin tumors. In addition to basic photoprotection guidelines, for SOTRs with multiple skin cancers or high-risk tumors, it is recommended to consider reducing immunosuppression or switching to mTOR inhibitors [43]. Furthermore, although the evidence is limited regarding the use of drugs such as acitretin [44] and nicotinamide [45,46], among others [47,48], different experts recommend specific chemoprophylaxis regimens to reduce the incidence of keratinocyte tumors in this high-risk subpopulation [49,50].

Among the main limitations of our study, we can include its retrospective nature. Additionally, the limited sample size and the low prevalence of poor prognostic outcomes (local recurrence or metastasis) may have affected the statistical power of our study in finding statistically significant differences, especially regarding metastasis rates and the characterization of risk factors for the development of poor prognosis events. Furthermore, because the hospital where this study was conducted is a reference center that receives complex cases from other institutions, there could have been a selection bias that may have overestimated the risk of local recurrence and metastasis observed in our high-risk population.

## 5. Conclusions

In this study, although we observed a slight increase in the rates of unfavorable skin cancer outcomes in our cohort of cutaneous squamous cell carcinomas in solid organ transplant recipients, these differences were not statistically significant. These findings could align with existing studies in the literature, suggesting that the immunosuppressive status does not constitute an independent risk factor for a poor prognosis. Indeed, SOTRs may be a population that exhibits a higher incidence of tumors and more advanced stages, which would primarily be what influences the prognosis of these patients. Additionally, we noted that clinical diameters greater than 2 cm and the depth of invasion of cSCC in SOTRs were associated with a poorer prognosis.

Further studies are needed to enable a more refined characterization of the risk of experiencing adverse outcomes in solid organ transplant recipients with cSCC. This enhanced risk stratification, particularly for subpopulations like immunosuppressed patients, would facilitate the better selection of candidates for adjuvant treatments and the intensification of follow-up protocols to ensure the optimal management of high-risk tumors.

## Figures and Tables

**Table 1:** Demographic and clinicopathological characteristics of the population of the study (*n* = 849).

Patient Characteristics	cSCC in Organ Transplant Recipients (*n* = 64) *n* (%) or Mean (SD)	cSCC in Immunocompetent Patients (*n* = 785) *n* (%) or Mean (SD)	*p*-Value
Sex			
Male Female	52 (81.2%) 12 (18.8%)	557 (71.0%) 228 (29.0%)	NS
Age at diagnosis (years)			
	64.50 (12.62)	79.71 (10.08)	<0.001
Type of community			
Urban Rural	22 (34.4%) 42 (65.6%)	362 (46.1%) 423 (53.9%)	NS
Personal history of skin cancer			
No NMSC Melanoma NMSC + melanoma	22 (34.4%) 42 (65.6%) 0 (0%) 0 (0%)	381 (48.5%) 393 (50.1%) 8 (1.0%) 3 (0.4%)	0.02
Organ transplanted			
Kidney Liver Cardiac Multivisceral	52 (81.3%) 3 (4.7%) 7 (10.9%) 2 (3.1%)		
Operating group			
Dermatology	24 (37.5%)	372 (47.4%)	
Plastic surgery	28 (43.7%)	304 (38.7%)	
Maxillofacial surgery	9 (14.1%)	50 (6.4%)	NS
Primary care	1 (1.5%)	31 (3.9%)	
Others	2 (3.1%)	28 (3.6%)	
Tumor Location			
Head and neck Upper limbs Lower limbs Trunk	48 (75%) 13 (20.3%) 1 (1.6%) 2 (3.1%)	659 (84.1%) 67 (5.5%) 38 (4.8%) 20 (2.6%)	NS
Primary SCC Major diameter (cm)			
	1.75 (1.55)	1.87 (1.52)	NS
Depth of invasion (mm)			
	4.58 (2.72)	4.64 (3.16)	NS
Clark level			
II III IV V	2 (3.5%) 19 (33.3%) 23 (40.4%) 13 (22.1%)	77 (11.9%) 161 (24.9%) 188 (29.1%) 219 (33.9%)	NS
Histologic differentiation			
Poor Moderate Well	7 (11.5%) 26 (42.6%) 28 (45.9%)	100 (13.0%) 336 (43.8%) 332 (43.2%)	NS
Perineural invasion			
Absent Present	57 (89.1%) 7 (10.9%)	724 (97.7%) 18 (2.3%)	NS
Lymphovascular invasion			
Absent Present	63 (98.4%) 1 (1.6%)	767 (97.7%) 18 (2.3%)	NS
Involvement of deep structures			
Absent Fascia Muscle Cartilage Bone	56 (87.5%) 4 (6.3%) 1 (1.6%) 1 (1.6%) 2 (3.1%)	685 (87.3%) 58 (7.4%) 23 (2.9%) 9 (1.1%) 10 (1.3%)	NS
Surgical margins status			
Negative Positive	48 (75%) 16 (25%)	612 (77.9%) 173 (22.1%)	NS
Adjuvant radiotherapy			
	4 (6.25%)	41 (5.2%)	NS
Local recurrence			
Absent Present	54 (84.4%) 10 (15.6%)	684 (87.1%) 101 (12.9%)	NS
Metastasis			
Absent Present	60 (93.8%) 4 (6.3%)	746 (95.0%) 39 (5%)	NS
Metastasis site			
Lymph nodes Distant	2 (3.1%) 2 (3.1%)	38 (4.8%) 1 (0.1%)	NS

cSCC: cutaneous squamous cell carcinoma; NMSC: non-melanoma skin cancer; NS: non-significant (*p* > 0.05); SD: standard deviation.

**Table 2:** Estimated cumulative probability of developing metastasis or local recurrence in cutaneous squamous cell carcinomas among solid organ transplant patients.

	6 Months	12 Months	24 Months
Local recurrence SOTR Immunocompetent	8% 5%	8% 9%	8% 12%
Metastasis SOTR Immunocompetent	3% 7%	4% 7%	5% 7%

**Table 3:** Results of univariate analyses conducted using the Cox regression model to assess the predictors of poor outcomes, including local recurrence and metastasis, for cutaneous squamous cell carcinomas in solid organ transplant patients.

Variable	HR	CI (95%)	*p*-Value
Sex	2.69	(0.6–10.8)	NS
Age (years)	1.01	(0.9–1.0)	NS
SCC location	0.06	(0.0–6.3)	NS
Tumor diameter (>2 cm)	3.06	(2.2–4.3)	0.001
Depth of invasion (>4 mm)	5.06	(1.07–2.8)	0.040
Clark level of invasion	3.7	(1.3–10.4)	0.011
Histologic differentiation	2.48	(0.8–7.4)	NS
Perineural invasion	2.69	(0.5–13.0)	NS
Limphovascular invasion	1.0	(0.0–7.3)	NS
Margin status	1.2	(0.3–5.17)	NS

HR: Hazard ratio; CI: confidence interval; NS: Non-significant (*p* > 0.05).

**Table 4:** Baseline characteristics of solid organ transplant recipients who developed a second cutaneous squamous carcinoma.

Solid Organ Transplant Recipients Patients	*n* (%)
Age at time of transplantation	
<50 years-old >50 years-old	2 (9.1%) 20 (90.9%)
Retransplant	
No Yes	19 (86.4%) 3 (13.6%)
Type of first cutaneous tumor after transplantation	
BCC SCC	1 (4.5%) 21 (95.5%)
Type of second cutaneous tumor after transplantation	
BCC SCC	3 (16.7%) 15 (83.3%)
Time to second skin tumor development (in years) Media (DS)	3.48 (3.19)
Number of BCC after transplantation: Median (IQ)	1 (5)
Number of cSCC after transplantation: Median (IQ)	4 (4)

SD: standard deviation; IQ: interquartile range; BCC: basal cell carcinoma, SCC: squamous cell carcinoma.

**Table 5:** Immunosuppression treatment in solid organ transplant recipients with cutaneous squamous cell carcinoma.

Immunosuppression Agents	*n* (%)	Treatment Duration (Years) Mean (sd)
Steroids	20 (90.9%)	16.61 (10.06)
Tacrolimus	16 (72.7%)	9.50 (5.41)
Mycophenolate mofetil	9 (40.9%)	8.91 (7.72)
Micofenolic acid	6 (27.3%)	4.41 (4.05)
Everolimus	7 (31.8%)	9.97 (8.36)
Sirolimus	4 (18.2%)	17.95 (13.78)
Cyclosporine	4 (18.2%)	17.90 (3.02)

## Data Availability

The data presented in this study are available on request from the corresponding author.
